# Adenocarcinoma of the seminal vesicles complicated by antineutrophil cytoplasmic antibody vasculitis: a case report and review of the literature

**DOI:** 10.1186/1752-1947-7-59

**Published:** 2013-03-01

**Authors:** Hazel Lote, Ethna Mannion, Terence Cook, Thomas Cairns, Philip Savage

**Affiliations:** 1 Medical Oncology, Imperial Hospitals NHS Trust, Charing Cross Hospital, London W6 8RF, UK

## Abstract

**Introduction:**

Adenocarcinoma of the seminal vesicles is a very rare malignancy, with less than 100 cases reported worldwide. It is documented to have a poor prognosis, with the majority of patients developing metastatic disease, most commonly in the prostate, bladder and rectum. Currently there is no standard treatment for metastatic disease and the limited reports of treatment with radiotherapy, chemotherapy and hormonal (anti-androgenic) therapy show that they are generally of modest benefit. The association between malignancy and an increased risk of autoimmune vasculitis has been demonstrated in a number of malignancies, but to date there have been no documented cases of adenocarcinoma of the seminal vesicles associated with anti-neutrophil cytoplasmic antibody vasculitis.

**Case presentation:**

In this report we describe the case of a 55-year-old Caucasian man with metastatic adenocarcinoma of the seminal vesicles. He previously had received chemotherapy treatment for advanced testicular cancer and later presented with hemospermia. He subsequently developed c-antineutrophil cytoplasmic antibody vasculitis requiring intensive immunosuppression and renal dialysis.

**Conclusion:**

Adenocarcinoma of the seminal vesicles is a rare diagnosis and our case is more unusual in that our patient previously had chemotherapy treatment for advanced testicular cancer and went on to develop severe antineutrophil cytoplasmic antibody vasculitis when diagnosed with metastatic seminal vesicle cancer. This case illustrates that autoimmune vasculitis can occur in any patient with malignancy and an early referral to the renal team combined with renal biopsy can assist in the earlier diagnosis and more successful management of these rare events. This case should be of interest to oncologists, renal physicians, urologists and general physicians who encounter patients presenting with hemospermia or vasculitis.

## Introduction

Adenocarcinoma of the seminal vesicles is a very rare malignancy, with fewer than 100 cases reported worldwide [1-7]. There have been no clear etiological factors demonstrated. The malignancy characteristically presents with either obstructive uropathy or hematuria with the imaging demonstrating tumor masses in the seminal vesicles. On radiology it is difficult to distinguish primary carcinoma of the seminal vesicles from a local invasion from prostate cancer. However, the immunohistochemistry of seminal vesicle carcinoma is characterized by positive staining for cancer antigen 125 (CA-125) and cytokeratin (CK)-7 and a lack of expression of membrane prostate-specific antigen, which helps differentiate the rare diagnosis of seminal vesicle carcinoma from the more frequent cases of prostatic cancer invading the seminal vesicles [4]. Serum CA-125 levels may be elevated in seminal vesicle carcinoma and may correlate with treatment response [6,7].

For local disease, surgery can allow long-term survival if there is no residual disease [1]. Radiotherapy has a role in the adjuvant setting if there are positive resection margins following surgery [1,6].

Because it is often detected late, adenocarcinoma of the seminal vesicles is documented to have a poor prognosis, with the majority of patients developing metastatic disease, most commonly in the prostate, bladder and rectum [2]. The lung may be a site of distant metastasis [1,6].

Currently there is no standard treatment for metastatic adenocarcinoma of the seminal vesicles. The limited reports of treatment with chemotherapy (with regimes including 5-fluorouracil plus leucovorin plus oxaliplatin) show that it has, at most, a modest benefit [1,2,6]. Hormonal (anti-androgenic) therapy appears slightly more promising, with some reports of patients surviving >24 months [1]. Treating with a combination of chemotherapy plus anti-androgenic therapy may have a role and has been reported in one case to give a time of 16 months until disease relapse.

The association between malignancy and an increased risk of autoimmune vasculitis has been demonstrated in a number of malignancies [8], but to date there have been no documented cases of adenocarcinoma of the seminal vesicles associated with antineutrophil cytoplasmic antibody (ANCA) vasculitis.

We describe the case of a 55-year-old man with adenocarcinoma of the seminal vesicles who subsequently developed cytoplasmic ANCA vasculitis requiring intensive immunosuppression and renal dialysis.

## Case presentation

A 55-year-old Caucasian man presented with an episode of hemospermia. Our patient had a complex urological history with bilateral inguinal hernias and undescended testes as a child. At the age of 11 years he underwent a right orchidopexy; but the left intra-abdominal testis could not be located during the operation. At the age of 26 years, our patient presented with a large mass in his abdomen, which was removed surgically and confirmed as a testicular cancer arising within an intra-abdominal testis. Postoperative treatment with cisplatin-based combination chemotherapy was delivered and regular check-ups showed no evidence of disease relapse.

Our initial investigations for hemospermia included a cystoscopy, which revealed a 9cm mass present within his bladder. The result of a biopsy of this mass was suggestive of adenocarcinoma of the seminal vesicle, because the tumor appeared to arise from the seminal vesicle epithelium. Computed tomography imaging revealed no evidence of metastatic spread. His serum prostate-specific antigen level was normal at presentation, but his serum CA-125 was 783kU/L. Following diagnosis, a referral was made to Charing Cross Hospital for specialist surgical input, resulting in a radical cystoprostatectomy and orchidectomy (required for local disease clearance) with the formation of an ileal conduit.

**Figure 1:**
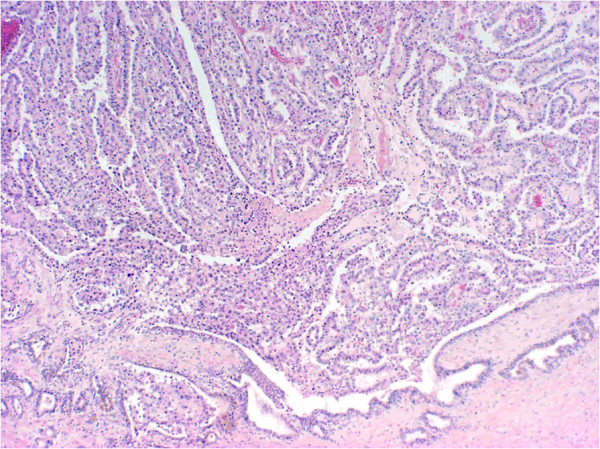
**Histopathology image of seminal vesicle adenocarcinoma.** Low power hematoxylin and eosin stain, ×4 objective, ×40 magnification, showing adenocarcinoma arising from the seminal vesicle (bottom right of picture).

Histopathology confirmed the diagnosis of adenocarcinoma of the seminal vesicle (Figure 1) with immunohistochemical studies positive for CK-7 and CA-125, while staining for prostate-specific antigenand CK-20 was negative. The tumor was noted to be widely infiltrative, involving the bladder, perivesical fat, prostate, prostatic urethra and ductus deferens, with additional pelvic lymph node metastases and a positive biopsy from an inoperable peritoneal deposit.

In view of the biopsy-proven distant metastasis (the peritoneal deposit), adjuvant radiotherapy was considered inappropriate and the initial management was of expectant observation. After three months observation, updated imaging demonstrated disease progression with malignant lymphadenopathy in his pelvis and a recurrence of the intra-peritoneal nodule combined with a rising CA-125 level.

Androgen withdrawal was commenced using goserelin, but after an initial response of five months, the tumor demonstrated evidence of progression with rising CA-125 levels and enlarging lymphadenopathy. Despite the serological and radiological progression, our patient remained asymptomatic and a decision regarding the role of chemotherapy treatment in disease palliation was deferred. Approximately six weeks later, our patient presented as an emergency with a short history of nausea, vomiting and diarrhea. The admission investigations showed his previously normal creatinine levels to now be elevated at 537μmol/L and urine testing revealed proteinuria >300mg/dL and hematuria with red cell casts. Imaging did not demonstrate an obvious cause for this rise in creatinine: ultrasound showed no hydronephrosis or evidence of obstruction, and computed tomography continued to show enlarging abdominal lymphadenopathy but was not significantly altered from the imaging that took place six weeks earlier.

**Figure 2:**
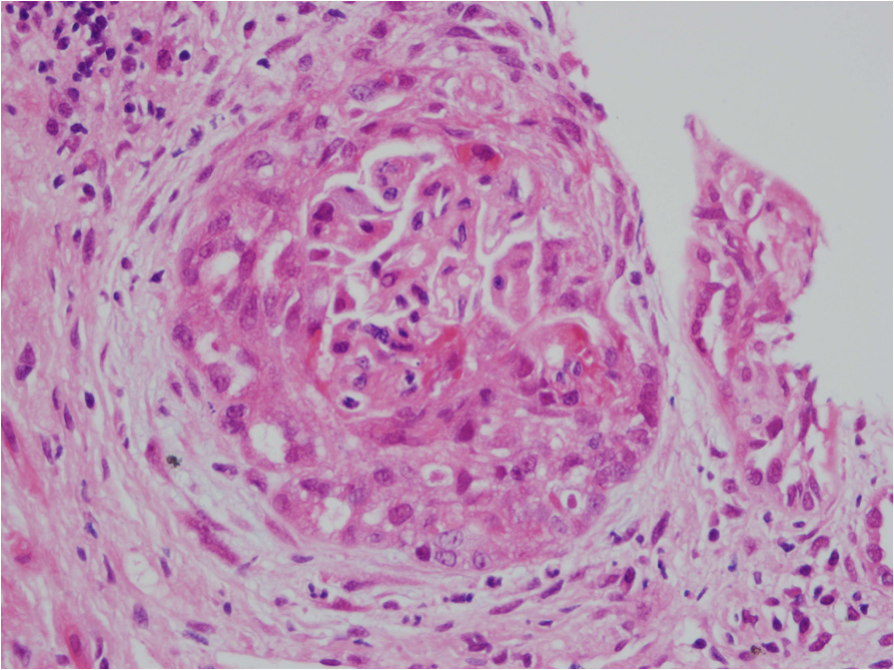
**Histopathology image showing antineutrophil cytoplasmic antibody vasculitis.** Renal biopsy showing glomerulus with circumferential cellular crescent.

A renal biopsy (Figure 2) showed pauci-immune crescentic glomerulonephritis with a segmental glomerular necrosis suggestive of ANCA-associated disease. A serum ANCA screen was ANCA IIF positive with a cytoplasmic ANCA pattern, while proteinase 3 and myeloperoxidase antibodies were negative.

Renal replacement therapy (hemodialysis) and immunosuppressive therapy was commenced with prednisolone, cyclophosphamide and rituximab but his renal function did not significantly recover. His glomerular filtration rate remained less than 10mL/min. A repeated renal biopsy performed in July 2011 after three months of therapy showed glomerular and tubulointerstitial scarring secondary to ANCA-mediated glomerulonephritis but no active ANCA-mediated disease.

An updated computed tomography scan performed at this point demonstrated significant tumor progression, with new sites of disease in his liver, bones and adrenal glands. In view of these findings and the challenges of delivering chemotherapy while on dialysis, attempts at disease palliation with chemotherapy were withheld. Our patient was referred to our community palliative care team for end-of-life care and died at home two months later.

## Discussion

Adenocarcinoma of the seminal vesicles is a rare diagnosis and this case is more unusual in that our patient had previously had chemotherapy treatment for advanced testicular cancer and went on to develop severe ANCA vasculitis when diagnosed with metastatic seminal vesicle cancer.

There is considerable evidence indicating that the survivors of chemotherapy treatment develop an enhanced risk of developing a second malignancy. A number of papers have indicated that the risk of developing a further solid tumor is in the order of one in three (compared with around one in five in the general population) and this increased incidence becomes apparent approximately five years after treatment [9,10]. For a series of 40,576 patients, the most common solid tumor second malignancies for men previously treated for testicular cancer were bladder and stomach cancers, but there were no reported cases of adenocarcinoma of the seminal vesicles. As a result, it is unclear if in our case the seminal vesicle malignancy was a true ‘second’ malignancy or simply a rare unlinked event.

The link between malignancy and vasculitis is well described but the etiology remains unclear [11]. It is believed to involve the abnormal production of proteins that bind to endothelial walls, molecular mimicry, and also defective apoptosis [12]. The most common malignancies linked to the development of vasculitis are hematological malignancies and lung cancer. Urological malignancies are less commonly linked with this event [11].

In our patient, the vasculitis became clinically evident approximately nine months after the diagnosis of metastatic seminal vesicle cancer and was linked to the presence of ANCA IIF autoantibodies with a cytoplasmic ANCA pattern. The mechanism of the development of ANCA vasculitis in cancer remains unclear, but several studies confirm that malignancy is associated with ANCA vasculitis [13].

The standard management of vasculitis is to use powerful immunosuppression combined with renal replacement therapy. A review of the literature suggests that ANCA vasculitis is more difficult to treat in patients with malignancy, as the vasculitis may be refractory to standard therapy. Resolution of the vasculitis is related to successful treatment of the underlying malignancy [14].

The development of chronic or recurrent vasculitis may represent an underlying malignancy. More importantly, the appearance of vasculitis can suggest recurrence or progression of a tumor. The overall outcomes for patients with severe vasculitis and malignancy are poor, with the most important predictor of a better prognosis being response of the underlying malignancy to treatment. Further large-scale studies are needed to evaluate the associations and interactions between vasculitis and malignancy [14].

In our patient, there was little scope to treat the underlying malignancy and the benefits for immunosuppression were limited because of the requirement for on-going dialysis. However, our case illustrates that autoimmune vasculitis can occur in any patient with malignancy. We recommend an early referral to the renal team combined with renal biopsy to assist in the earlier diagnosis and more successful management of these rare events.

## Conclusions

Adenocarcinoma of the seminal vesicles is a rare diagnosis and may subsequently lead to cytoplasmic ANCA vasculitis requiring intensive immunosuppression and renal dialysis. Appropriate subspecialty referral is warranted.

## Consent

Written informed consent was obtained from the patient’s next of kin for publication of this case report and accompanying images. A copy of the written consent is available for review by the Editor-in-Chief of this journal.

## Abbreviations

ANCA: Antineutrophil cytoplasmic antibody; CA-125: Cancer antigen 125; CK: Cytokeratin.

## Competing interests

The authors declare that they have no competing interests.

## Authors’ contributions

HL drafted the manuscript and performed the literature review. EM performed the histological examination of the seminal vesicle. TC performed the histological examination of the kidney. TC organized the renal biopsy and contributed information to the case. PS revised the manuscript critically and was a major contributor in writing the manuscript. All authors read and approved the final manuscript.
